# BNP as a Major Player in the Heart-Kidney Connection

**DOI:** 10.3390/ijms20143581

**Published:** 2019-07-22

**Authors:** Ryuji Okamoto, Yusuf Ali, Ryotaro Hashizume, Noboru Suzuki, Masaaki Ito

**Affiliations:** 1 Department of Cardiology and Nephrology, Mie University Graduate School of Medicine, 2-174 Edobashi, Tsu, Mie 514-8507, Japan; 2 Department of Pathology and Matrix Biology, Mie University Graduate School of Medicine, 2-174 Edobashi, Tsu, Mie 514-8507, Japan; 3 Department of Animal Genomics, Functional Genomics Institute, Mie University Life Science Research Center, 2-174 Edobashi, Tsu, Mie 514-8507, Japan

**Keywords:** natriuretic peptide, cardiorenal syndrome, vasopressor, vasodilator, kidney, medulla, renin-angiotensin-aldosterone system

## Abstract

Brain natriuretic peptide (BNP) is an important biomarker for patients with heart failure, hypertension and cardiac hypertrophy. Although it is known that BNP levels are relatively higher in patients with chronic kidney disease and no heart disease, the mechanism remains unknown. Here, we review the functions and the roles of BNP in the heart-kidney interaction. In addition, we discuss the relevant molecular mechanisms that suggest BNP is protective against chronic kidney diseases and heart failure, especially in terms of the counterparts of the renin-angiotensin-aldosterone system (RAAS). The renal medulla has been reported to express depressor substances. The extract of the papillary tips from kidneys may induce the expression and secretion of BNP from cardiomyocytes. A better understanding of these processes will help accelerate pharmacological treatments for heart-kidney disease.

## 1. Introduction

The Brain natriuretic peptide (BNP), which is a component of the natriuretic peptide (NP) system and also known as B-type NP, is mainly secreted from the cardiomyocytes in response to cardiac stretch and ischemia, and plays an important role in cardiorenal protection [1,2,3,4]. The renoprotective effects of BNP include the inhibition of sodium reabsorption in the proximal tubule and the distal nephron, and the improvement of the glomerular filtration rate (GFR) and renal plasma flow (RPF) with respect to vasodilatation by inhibiting multiple plasma vasoconstrictors [5,6,7]. Furthermore, BNP infusion inhibits the cardiac and renal sympathetic tones [8] and the renin-angiotensin-aldosterone system (RAAS) [9], in addition to decreasing the endothelin release [10]. NPs mediate their functions through interactions with specific surface receptors on target cells. At present, there are three distinct natriuretic peptide receptors (NPR) that have been reported and which include NPR-A, NPR-B, and NPR-C. NPR-A and NPR-B stimulate guanylyl cyclase, mediating their effect via the activation of the second messenger, cyclic guanosine monophosphate (cGMP). In the kidney, NPs cause a relaxation of mesangial cells, which increases GFR and reduces fractional sodium reabsorption in the renal tubules [7,11,12,13].

Congestive heart failure (CHF) is a complex syndrome characterized by sodium and water retention through the activation of different neurohormonal systems, such as the RAAS and the sympathetic nervous system (SNS), and also importantly, the NP system. Several experimental and clinical studies have implicated BNP in the pathophysiology of the unbalanced cardiorenal axis in CHF. In CHF patients, BNP plasma levels are in excess of 100 pg/mL [14]. Due to BNP’s longer half-life, it has been shown to have greater stability and offer a better understanding of the disease progression diagnostically as compared to atrial NP (ANP) in terms of improving cardiovascular function [15,16,17,18]. BNP plasma levels are also elevated in chronic kidney disease (CKD) patients. Underlying causes include renal dysfunction, diminished neprilysin (NEP) activity in the kidney and associated cardiovascular pathophysiology. Previous studies reported finding markedly high plasma BNP levels in patients with renal impairments [19,20,21].

Growing evidence suggests that in CHF, coronary artery disease and/or left ventricular hypertrophy etc., are increasingly associated with CKD patients. More recently, the cardiorenal syndrome (CRS), which is a complex pathophysiological condition that involves an association between acute heart failure (AHF) or CHF and renal impairment, has received much more attention. However, this condition is more than just a simultaneous cardiac and renal disease [22]. A recent French prospective study on 507 AHF patients demonstrated that BNP and the BNP prohormone were higher in AHF patients with renal dysfunction (CRS patients) as compared to those with normal renal function [23].

BNP acts as a compensating agent in the early stages of disease progression by inducing natriuresis and dieresis, and reducing RAAS and SNS. Similar to that seen with severe disease states like HF or CRS and despite high levels, endogenous BNP becomes resistant and is no longer able to compensate for volume overload in such cases. Thus, the supportive role of BNP in counteracting the unwanted effects of activated RAAS and SNS in these diseases provides the rationale for using this peptide as a potential therapeutic agent [13,24]. As it has been reported that the renal medulla expresses depressor substances, an extract of the papillary tips from kidneys might be able to induce the expression and secretion of endogenous BNP from cardiomyocytes [25]. A better understanding of these processes could potentially accelerate pharmacological treatments for CRS. Here, we review the functions and the roles of BNP in the heart-kidney interaction. In addition, we discuss the relevant molecular mechanisms for the protective effect of BNP against CKD and HF, especially in terms of the counterparts of the RAA system.

## 2. Biochemical Characteristics of BNP

Human BNP mRNA is translated to preproBNP of 134 amino acids, from which proBNP of 108 amino acids is processed and cleaved by the serine proteases corin and/or furin, yielding a biological inactive amino-terminal fragment, 76-amino acid proBNP (NT-proBNP) and an active carboxy-terminal fragment, 32-amino acid BNP [6,26]. It has been considered that, after maturation, both NT-proBNP and BNP are secreted at a 1:1 molar ratio from the heart. However, recent studies have shown that a certain amount of proBNP is also secreted without its cleavage from the heart [27]. Therefore, we should take into consideration the possibility that the increase in NT-proBNP is partly due to the elevation of proBNP when we see patients with an elevation of NT-proBNP. In contrast, BNP is increased without additional cardiac stress in patients treated with angiotensin-receptor/Neprilysin inhibitor (ARNi) due to its inhibitory effects of BNP degradation. In this review, we will not discuss them in detail here. Both NT-proBNP and BNP are equally useful in the differential diagnosis of heart failure [28,29], although the NT-proBNP and BNP assay allows for complementary information during ARNi treatments [30]. The gene structure and the post-translational processing of BNP and the diversity of circulating BNP-related peptides have been reviewed in detail [2,6,31].

## 3. The Function of BNP in Kidneys

It has been well established that BNP plays important roles in the kidneys, and provides multiple beneficial effects involving renal function [3]. Abnormalities or alterations of this system may contribute to renal impairment including tubular damage as a consequence of other CV disorders [32]. On the other hand, BNP plasma levels are affected by renal function, although the mechanism remains unclear. Thus, during renal failure, these are not considered to be an ideal choice as hemodynamic biomarkers [33,34]. The elevated levels of BNP may be the result of an increased cardiac release in CKD patients. In CKD patients, the increase in circulatory blood volume, the elevation in BP due to volume overload and arterial stiffness, and the cardiac hypertrophy and HF etc., can contribute to the elevation in BNP. The elevation of BNP in CKD patients is partly due to the impaired clearance of BNP from the kidneys.

**Figure 1:**
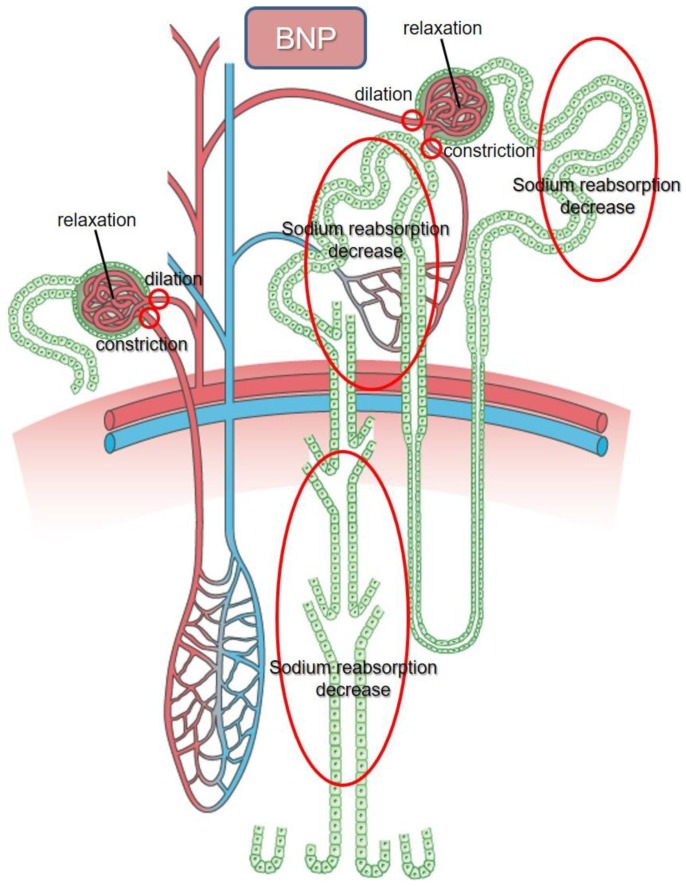
The interaction site of BNP in the nephron. BNP: brain natriuretic peptide.

The functions of NPs are mediated by their interactions with specific surface receptors on the target cells. In the kidney, BNP increases GFR by relaxing the mesangial cells and inhibits the tubular fractional reabsorption of sodium [7,35] (Figure 1). BNP also decreases vascular resistance by relaxing vascular smooth muscle cells, while it has no effect on vascular permeability, unlike ANP. 

Table 1 shows a sequence of pioneer studies that examined the injection of BNP in humans [5,9,35,36,37,38,39,40,41,42,43,44,45,46,47,48,49,50,51,52]. Among them, Jensen et al. performed a well-organized study of BNP infusion in healthy men in 1998 [35]. The maximum concentration in the plasma that was reached was 199 pmol/L (688.6 pg/mL) after a dose of 4 pmol/kg/min for 60 min. The authors observed that there was an increase in the urinary flow rate and GFR (about +60% and +5% respectively) and that there was inhibition of the renin secretion (−24%). In addition, the fractional excretion of sodium was increased (+140%). The fractional reabsorption of sodium was decreased under the infusion (30 to 60 min after the start of infusion). The measurement of the clearance using lithium, which is assumed to only be reabsorbed in the proximal tubule and to the same degree as sodium and water, demonstrated that the tubular site of action occurred both in the proximal tubules (−7%) and distal nephron (−5%). There were no changes in the blood pressure, heart rate and aldosterone concentration. There was a decrease in the ANF concentration (−16%). These results suggest that the infusion of BNP within the physiological range, which can be observed in patients with HF, induces an increase in the GFR and the inhibition of sodium excretion, which leads to an increase in both the urine volume and the sodium excretion without affecting the blood pressure and heart rate in healthy subjects. These results are similar to other studies in terms of urination and sodium excretion irrespective of the minor differences in the protocol and the results (Table 1). Sodium reabsorption appears to decrease in the distal nephron earlier than that observed in the proximal tubule [41]. Interestingly, a trial of BNP infusion (2 pmol/kg/min for 60 min) in patients with heart failure and reduced ejection fraction (HFrEF) due to previous myocardial infarction or dilated cardiomyopathy showed that the impaired natriuretic response due to the reduced responsiveness in the distal nephron in patients with HFrEF was comparable to that found in healthy control subjects [5] and the results reported in another previous study [41]. This finding may be of interest, as this is in line with another previous report that found that a reduced reabsorption of sodium occurs in the distal nephron earlier than in the proximal tubules in patients with mild CKD [53]. In the distal nephron, the inner medullary collecting duct is the most prominent target of ANP [13]. It has been reported that BNP is colocalized with ANP in the distal tubules, while CNP is observed in the proximal tubules in human kidneys [54]. Thus, the adaptive recovery from impaired reabsorption of sodium in the distal nephron and feasibility for lowering the blood pressure suggest these can be therapeutic targets if we can determine the range of nesiritide in patients with CHF or/and CKD [55,56]. Moreover, as the NPRs are predominantly expressed in the distal part of the renal tubules [57], this can be another possible reason for their impaired natriuretic effect in advanced HF in which the proximal reabsorption of sodium is greatly enhanced [58]. In an experimental CHF model, BNP enhances the renal diuretic and natriuretic actions of loop diuretics while at the same time it also reduces the diuretic-induced aldosterone production [59].

BNP infusion may play an important role in preventing the development of CKD. McKie et al. recently examined BNP’s role in the pathophysiology of cardiorenal dysfunction and found that in asymptomatic systolic HF patients, chronic subcutaneous BNP therapy for 12 weeks improved renal function along with favourable hemodynamic effects in response to volume expansion [60]. A recent study also investigated the prophylactic effects of early BNP administration on contrast-induced nephropathy (CIN) in CKD patients undergoing elective percutaneous coronary intervention or coronary angiography [61]. BNP effectively decreased the incidence of CIN in patients with CKD, as was shown by the improved estimated GFR, cystatin C, and serum creatinine compared to the control group. In addition, the BNP group showed a faster recovery. Thus, exogenous BNP served as a prophylactic agent for attenuating the CIN incidence in CKD patients [61]. A retrospective study analyzed the effect of nesiritide on renal function and its clinical safety in 328 patients with decompensated HFpEF (dHFpEF) and concluded that nesiritide can be safely administered without negatively impacting the long-term renal function in these patients [56]. The GFR and creatinine remained stable at 1-month post-nesiritide infusion, whereas there was a significant deterioration of kidney function (GFR and creatinine) observed in the control subjects. In addition, their multivariate analysis showed that nesiritide was an important predictor of renal function at 1 month [56].

The vascular effects of NPs are site-specific [62]. Importantly, in the kidney’s vasculature, while NPs relax the afferent arterioles by acting as vasodilators, they act as vasoconstrictive agents on the efferent arterioles, thereby causing the GFR to be increased [63]. Therefore, since it is presumed that the renal blood flow may increase or decrease or even remain unchanged in response to exogenous NPs, it can be accompanied by a similar discrepant result regarding the renal plasma flow [13].

In CKD patients, impaired renal function restricts the use of NPs, as plasma BNP levels are elevated to ~200 pg/mL in CKD patients without HF. Whether these elevated BNP levels in CKD promote the activation of the NP system and have an effect on the target organ still remains unclear. Downregulation of NPR-A and upregulation of NPR-C expression in the renal medulla and renal cortex, respectively, may be responsible for the resistance of NPs including BNP in CKD, thereby resulting in its limited use in treating CKD patients with HF [64,65].

## 4. BNP Clearance from Kidneys

BNP clearance is associated with two major pathways. The first involves binding to the NPR-C receptor, while the second one is involved with the degradation by neprilysin (NEP), a zinc metallopeptidase. NPR-C, which is devoid of any guanylyl cyclase activity and coupled to the adenylyl cyclase inhibition [66], plays an important role as a clearance receptor for BNP in addition to ANP and CNP. In humans, it has been demonstrated that NPR-C mRNA is expressed in a variety of tissues such as the atria, kidney, lung, mesentery, placenta, adrenal, heart, cerebral cortex, cerebellum and aortic smooth muscle and endothelial cells [67,68]. ANP has been shown to be degraded mainly in the lung, liver and kidney. The binding ability of BNP with NPR-C in these organs is much weaker than ANP and CNP [69], which suggests a diminished removal of BNP by NPR-C-mediated internalization, and a long half-life due to the smaller amount of degradation. BNP is also degraded by a neutral endopeptidase known as NEP, which is mainly expressed at high levels at the luminal side of the renal proximal tubules in the kidneys [70] in addition to other tissues such as the heart, lungs, liver and vascular smooth muscle and endothelial cells. NEP extensively degrades BNP in the rat renal membrane in collaboration with a renal protease. In the human renal membrane, however, not all BNPs are degraded by NEP [71]. Moreover, the NEP mediated degradation of BNP is slower [72]. These results suggest that it is difficult to clear BNP from the kidneys in humans. Despite the fact that BNP degradation is mediated by both the NPR-C and NEP pathways, the precise role of each process with regard to the BNP concentrations remains undetermined [72]. In addition to NEP, BNP has also been reported to be degraded by dipeptidyl peptidase-4 and insulin-degrading enzyme [73,74]. In CHF patients, a marked decrease in urinary excretion of NPs was observed in conjunction with elevated plasma levels [75]. However, another report showed that there was an increase of NT-proBNP in fresh urine from HF patients [76].

## 5. BNP System vs. RAA System

**Figure 2:**
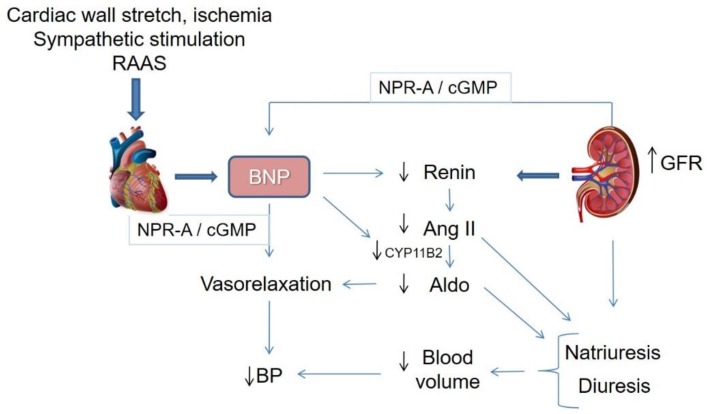
BNP as a counterregulatory system of the renin angiotensin II aldosterone system (RAAS). Aldo, aldosterone; Ang II, angiotensin II; BP, blood pressure; BNP, brain natriuretic peptide; CYP11B2, cytochrome P450 family 11 subfamily B member 2 (aldosterone synthase); cGMP, cyclic guanosine monophosphate; GFR, glomerular filtration rate; NPR-A, natriuretic peptide receptor A.

Sympathetic stimulation activates the RAAS, including the augmentation of angiotensin II (Ang II) and aldosterone production, as a result of the elevation of vasopressin and norepinephrine plasma levels, as well as the secretion of renin. SNS and RAAS augment the cardiac output by increasing heart rate, contractibility, preload and afterload at the expense of increased oxygen consumption [77]. Inhibiting the SNS through beta-blockers and antagonizing the RAAS through the angiotensin-converting enzyme (ACE) inhibitors or angiotensin receptor blockers (ARBs), and mineralocorticoid receptor antagonists, may be insufficient for certain neurohormonal abnormalities [78]. As a response to volume overload or cardiac stretching, secretion of BNP from the cardiac chambers manifests the heart as one of the endocrine organs in order to maintain the salt balance by interacting with the RAAS, SNS and the kidneys (Figure 2). Continual or excessive activation of the RAAS and SNS leads to the development of CHF. Furthermore, BNP is also activated in order to resist the actions attributed to the diuretic, natriuretic and vasorelaxant effects of BNP. As a result, this counterbalances the neurohormonal activation in CHF or hypertension leading to beneficial effects, such as natriuresis, vasodilation, and anti-cardiac remodelling (Figure 2) [79,80,81].

BNP has been shown to directly inhibit renin production from the kidney before affecting RBF or GFR [82], similar to ANP [83]. This result indicates that BNP directly inhibits the tubuloglomerular feedback response that is activated by salt over intake.

Experimental studies using rat cardiomyocytes reported that both endogenous and exogenous BNP reduced aldosterone synthase (CYP11B2) mRNA expression, which may lead to the inhibition of the RAAS and attenuation of cardiac hypertrophy and fibrosis [84]. Furthermore, in cultured primary human adrenocortical cells, it has been reported that BNP opposed the Ang II-stimulated biosynthesis of aldosterone due to decreased expression of both CYP11B2 and CYP11B1, which are the most important synthetic enzymes of aldosterone [85]. In contrast, BNP failed to directly inhibit the production of catecholamine and the synthesis of tyrosine hydroxylase, a dopamine synthetic enzyme, in rat adrenal pheochromocytoma cells [86]. Infusion of BNP induced a sympatho-inhibitory effect in normal subjects and inhibited renal sympathetic nervous activity in patients with CHF but not in healthy subjects [8]. However, the findings of another study that found that there were reductions in systemic and right-sided cardiac pressures in HF patients infused with BNP without any changes in the renin, aldosterone and norepinephrine plasma levels suggests that there is a RAAS or SNS independent direct vasorelaxant effect of BNP [47]. Both systems appear to influence each other, with BNP counteracting RAAS by inhibiting the renin secretion and CYP11B2 expression through cGMP, while the RAAS blockade, in turn, activates BNP. This suggests these may exert a synergistic effect in HF [87]. Thus, NP clearly interacts with the RAAS, and is inversely correlated with the plasma Ang II levels in certain physiologic conditions [88]. Furthermore, since the plasma levels of both hormonal systems are augmented in HF, this suggests that they counterbalance each other [89].

## 6. NPs Augmentation Combined with RAAS Blockade: Dual-Acting Angiotensin-Receptor/Neprilysin Inhibitors (ARNi)

Although BNP is progressively activated in HF, its response may often be insufficient to counteract the sodium retention and vascular constriction due to activation of RAAS and SNS. Therefore, more thorough approaches were undertaken both experimentally and clinically in an attempt to minimize the dysregulation of these neurohormonal systems. Ongoing strategies in promoting NP include synthesis or using agonists to increase its bioactivity and inhibition of NEP to reduce its catabolism [90,91]. Nesiritide, a recombinant BNP approved by the US Food and Drug Administration (FDA) in 2001, has been shown to promote clinical improvements in the management of CHF [92]. However, it has been reported to worsen renal function and increase the mortality rate in a meta-analysis [93]. Moreover, the severe hypotension and short half-life made these agents, including nesiritide, carperitide and ularitide, clinically imperfect. NEP inhibitors (NEPi) alone leads to activation of RAAS and attenuated Ang II degradation. Again the NEPi and ACE inhibitor combination predisposes a high risk of angioedema [94]. Therefore, the use of an ARB would be the optimal method of RAAS inhibition for use with NEPi. The first angiotensin receptor–neprilysin inhibitor (ARNI, LCZ696), developed by combining an ARB (Valsartan) with a NEPi (Sacubitril), was a major advance in the therapies for HF [95,96]. The combination of sacubitril and valsartan augments the beneficial effects of NPs and inhibits the harmful effects of Ang II. It preserves the ACE mechanism for bradykinin degradation and protects from angioedema formation [97]. Animal studies have shown that the NEPi and RAAS inhibitor combination reduced proteinuria and prevented kidney damage [98], also improved cardiac remodelling, fibrosis, and hypertrophy [99]. The recent UK HARP-III (United Kingdom Heart and Renal Protection-III), study on 414 CKD patients with an eGFR 20 to 60 mL/min/1.73 m^2^ has demonstrated that over 12 months, the combination of sacubitril and valsartan was well tolerated and had similar effects on kidney function and albuminuria compared to ARB irbesartan, with a BP and cardiac biomarker lowering effect, suggested that this combination could have the potential for reducing cardiovascular risk in CKD [100].

LCZ696 lowered BP more effectively than valsartan in hypertensive patients [101]. The PARAMOUNT (Prospective comparison of ARNI with ARB on Management Of heart failUre with preserved ejectioN fracTion, HFpEF) trial demonstrated that compared to valsartan, LCZ696 improved the overall clinical status and decreased atrial pressure and elevated GFR as well [102]. In HF patients with reduced ejection fraction (HFrEF), LCZ696 was reported to be more effective than enalapril in reducing hospitalization and cardiovascular and sudden death, preventing HF progression and improving renal function, as well as quality of life, in the Prospective comparison of ARNI with the ACE inhibitor to Determine Impact on Global Mortality and Morbidity in Heart Failure (PARADIGM-HF) trial [103,104,105,106]. Based on the myriads of favourable results of this trial, LCZ696 has been approved by the FDA in 2015 for treating HFrEF. ARNI has been shown in several studies including the PARADIGM-HF trial, to improve kidney function compared with RAAS inhibitor in HF [102,106,107]. Recently, an experimental study using a mouse myocardial infarction (MI) model and an in vitro mouse peritoneal macrophage, demonstrated that LCZ696 was associated with a better balance between the RAA and NP systems, and attenuated cardiac rupture following MI, suggesting that LCZ696 by its dual regulating mechanisms inhibited the inflammation and degradation response of macrophages and that early treatment with LCZ696 might have a cardioprotective effect after MI [108].

In spite of having numerous benefits of ARNi so far, however, some concerns raised that limits its clinical use, such as the elevation of bradykinin levels with ARB, the development of angioedema [109], and the chance of inducing Alzheimer disease by blocking the breakdown of amyloid-β [110]. Importantly, since sacubitril is predominantly excreted in the kidney in patients with renal impairment, it may accumulate and induce the development of hypotension particularly in patients with borderline BP [74].

## 7. Vasopressor and Vasodepressor Derived from Kidneys

**Figure 3:**
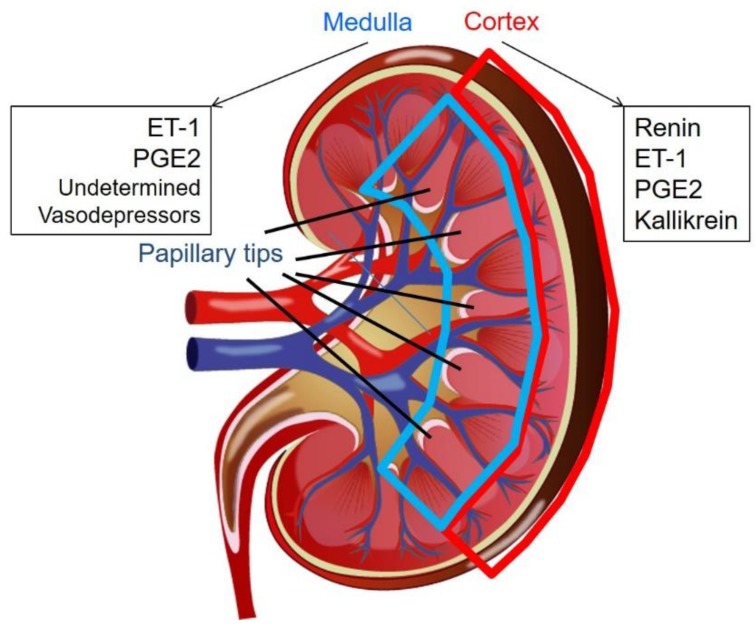
The vasopressors and vasodepressors derived from the kidneys. ET-1, endothelin 1; PGE2, prostaglandin E2.

Kidneys play an important role in regulating blood pressure. In addition to volume control via urination with balanced water and electrolytes excretion, the kidneys possess several substances that are used to control blood pressure [111] (Figure 3). The most well-known substance is derived from the renal cortex. Tigerstedt and Bergman demonstrated that the injection of cortical extracts from rabbit kidneys caused a blood pressure elevation in the recipient rabbits, which led to the subsequent discovery of renin, a powerful vasopressor [112].

Endothelin-1 (ET-1), a 21 amino acid vasoconstrictor, is also produced by the endothelial cells, tubular cells and inner medullary collecting duct cells [113]. In physiological states, increased salt intake induces the tubular production of ET-1, which inhibits epithelial sodium channels and causes a reduced sodium reabsorption rate that leads to natriuresis. However, in pathophysiological states, ET-1 has been considered to be a vasopressor due to the development of glomerular and interstitial kidney and vascular diseases [114].

Prostaglandin (PG) is a lipid mediator involved in a variety of physiological and pathological processes in the kidneys [115]. PGE2 is the most abundant renal arachidonic acid metabolite. PGE2 is produced by the microsomal PGE synthase in the macula densa, distal convoluted tubule, collecting duct and renal medullary interstitial cells (RMIC) [116]. Although there are still conflicting conclusions, PGE2 is generally considered to be a vasodepressor due to its diuretic effect [117].

Tissue kallikrein is mainly expressed in the submandibular gland, pancreas and kidneys. Kallikreins are primarily produced in the distal convoluted tubule [118,119], the cortical collecting tubule [119,120], and possibly in the collecting tubule in the inner medulla [120]. Tissue kallikrein releases kinins from low and high molecular-weight kininogen. Kinins are inactivated kininases and the best-known kininase is the angiotensin-converting enzyme (ACE). In humans, tissue kallikrein releases Lys-bradykinin (kallidin). The kallikrein-kinin system increases the renal blood flow leading to more urination, water and sodium excretion [121] in collaboration with its direct effect on the distal nephron [122]. It has been reported that a reduced urinary kallikrein is associated with the development of hypertension [123,124,125]. Thus, kallikrein is considered to be a vasodepressor.

It has been reported by several independent groups that the renal medulla possesses a depressor substance that acts as a counterpart to RAAS [126,127]. This depressor agent is produced from RMICs. RMICs contain intracellular endocrine granules, which are reduced in size in proportion to blood pressure and renal blood flow [128]. Although the inclusions have not been correctly identified, electron microscopy studies have revealed that the contents consist of free fatty acids and PGs. However, the depressor substance does not seem to be a PG, nitric oxide or a platelet-activating factor [126,129]. The renal papillary RMICs have abundant granules when a kidney is clipped, and they have been shown to degranulate after unclipping in a one-kidney and one-clip hypertensive rat model [130]. It remains unknown whether the renal papillary tip contains the same substance the researchers have been trying to identify in the renal medulla, as most researchers did not distinguish between the papillary tip and the entire inner medulla.

## 8. Renal Papillary Tip May Contribute to the Expression of BNP in Cardiomyocytes

We recently developed a BNP reporter mouse carrying tdTomato under the mouse promoter of the 1136-bp fragment of the mouse *NPPB* gene from −1000 to +136 and demonstrated that this promoter was specifically activated in the papillary tips of the kidneys and was not accompanied by the BNP mRNA expression [25]. No evidence has been found that shows the existence of BNP isoforms or other nucleotide expressions apart from BNP and tdTomato. After the treatment with the extract from the renal papillary tip, both the expression and the secretion of BNP unexpectedly increased in the primary cultured neonatal cardiomyocytes. Although it is possible that artefacts due to contamination could occur, we found that there was no change in the expression of Ang II, ET-1, and type A, B and C NPs between the papillary tip and other portions of the kidneys. Even though its mechanism remains unknown, we initially evaluated elderly female mice as ageing and the female sex contribute to the expression of BNP in both normal subjects and patients with CHF [131,132,133,134]. However, we observed a similar activation of the BNP promoter in the papillary tips from young adults and/or male adult mice, although this was not recognized in neonatal mice [25].

The pBNP-tdTomato-positive cells were interstitial cells and were not proliferative. The papillary medulla has been reported to possess the ability to decrease blood pressure due to its vasodilatory activity [126,127]. To evaluate this activity of the papillary medulla from the kidneys, we injected an extract of the papillary tip intraperitoneally into stroke-prone spontaneously hypertensive rats (SHR-SPs). Intraperitoneal injection of the papillary extract reduced blood pressure from 210 mmHg to 165 mmHg and this was accompanied by an increase in serum BNP and urinary cGMP production in SHR-SP rats. Furthermore, the treatment with the papillary extract from rats with heart failure due to myocardial infarction significantly induced BNP expression in cardiomyocytes [25].

## 9. Conclusions

BNP plays an important role as a major player in the heart-kidney connection via its inhibitory effect on the RAAS, especially in the heart and the kidneys. Kidneys possess several substances involved in regulating the blood pressure in addition to volume control via urination. Furthermore, the papillary tips may play important roles in regulating the BNP expression from cardiomyocytes. Additional investigations will need to be undertaken in order to determine the relationship between the renal depressor system and BNP regulation, especially in terms of cardiovascular diseases, such as heart failure, hypertension and CKD.

## Figures and Tables

**Table 1:** The effects of brain natriuretic peptide (BNP) infusion on renal function, the renin angiotensin II aldosterone system and hemodynamics in normal subjects and patients with heart failure and hypertension.

Study/Reference	Dosage of BNP pmol/kg/min	GFR	RPF	Urine Volume	Urine Na	Urine cGMP	Urine Aldo	Plasma cGMP	PRA	AngII	Plasma Aldo	MAP	HR	CO	SVR	PCWP
**Normal Subjects**																
McGregor. *J. Clin. Endocrinol. Metab*. 1990 [36]	2				↑↔ *	↑			↓		↓	↔	↔			
Yoshimura. *Circulation*. 1991 [37]	30			↑	↑			↑	↔		↓	↓	↑	↑	↓	↓
Holmes. *J. Clin. Endocrinol. Metab*. 1993 [9]	2			↔	↑	↑		↑	↓↔ *		↓	↔	↑			
Cheung. *Clin. Sci*. 1994 [38]	0.4	↔		↔	↑							↔	↔			
Florkowski. *Am. J. Physiol*. 1994 [39]	2(+ANP(2)) **	↔			↑	↑		↑	↔	↔	↔	↓	↔			
La Villa. *J. Clin. Endocrinol. Metab*. 1994 [40]	4	↑	↑	↑	↑	↑			↔		↔	↔	↔	↔	↔	
La Villa. *Hypertension*. 1995 [41]	0.25 and 0.5	↔	↔	↔	↑	↑	↓		↓			↔	↔			
Lazzeri. *Cardiology*. 1995 [42]	4, 8, 10, 12							↑	↔			↔	↑	↔	↔	
Hunt. *J Clin. Endocrinol. Metab*. 1996 [43]	2	↔		↑	↑			↑	↓↔ *	↓	↓	↔	↑			
Yasue. *J Card. Fail*. 1996 [44]	30	↑		↑	↑			↑				↓	↑		↓	↓
Jensen. *Am. J. Phy*.1998 [35]	1, 2 and 4	↑	↓	↑	↑	↑		↑	↓	↔	↔	↔	↔			
Jensen. *Clin. Sci*. 1999 [5]	2	↑	↓ ***	↔	↑	↑		↑	↓	↔	↔	↑	↔			
van der Zander. *Am. J. Physiol*. 2003 [45]	4	↑	↔	↑	↑			↑				↔	↔	↓↔ *	↔	
**Summarized**		↑	↔	↑	↑	↑		↑	↓↔	↔	↓	↔	↔	↔	↔	↓
**Patients with Heart Failure**																
Yoshimura. *Circulation*. 1991 [37]	30			↑	↑			↑	↔		↓	↓	↑	↑	↓	↓
Marcus. *Circulation*. 1996 [46]	1 to 30	↔		↑	↑			↑				↓	↓	↑	↓	↓
Yasue. *J. Card. Fail*. 1996 [44]	30	↔		↑	↑			↑				↔	↔		↓	↓
Lainchbury. *HTN* 1997 [47]	3.3	↔		↔				↑	↔		↔	↓	↔	↔	↓	↓
Abraham. *J. Cardiac. fail*. 1998 [48]	7.5, 15	↔	↔	↔	↔			↑	↔		↓	↓	↔	↑	↓	↓
Jensen. *Clin. Sci*. 1999 [5]	2	↔	↔	↔	↑	↑		↑	↓	↔	↔	↑	↔			
Wang. Am. *J. Trans. Res*. 2016 [49]	A bolus followed by 2 to 6 for 72 h	↓										↓				
**Summarized**		↔	↔	↑	↑	↑		↑	↔	↔	↓	↓	↔	↑	↓	↓
**Patients with Hypertension**																
Richards. *J. Hypertension*. 1993 [50]	2	↔		↑	↑	↔		↑	↔ ^#^		↓	↔	↔			
Lazzeri. *Am. J. Hypertension*. 1995 [51]	4	↑		↑	↑	↑			↔			↔	↔	↔	↔	
Pidgeon. *Hypertension*. 1996 [52]	2	↑		↑	↔			↑	↓		↓	↓	↑			
**Summarized**		↑		↑	↑			↑	↔		↓	↔	↔			

ANP, atrial natriuretic peptide; cGMP, cyclic guanosine monophosphate; CO, cardiac output; GFR, glomerular filtration rate; HR, heart rate; MAP, mean arterial pressure; PCWP, pulmonary capillary wedge pressure; PRA, plasma renin activity; RPF, renal plasma flow; SVR, systemic vascular resistance. * marginal significance. ** BNP(2)+ANP(2) vs ANP(2) alone. *** after the infusion. ^#^ Plasma renin concentration.

## Abbreviations

ANP	atrial natriuretic peptide
BNP	brain natriuretic peptide
cGMP	cyclic guanosine monophosphate
CHF	congestive heart failure
CKD	chronic kidney disease
CRS	cardiorenal syndrome
GFR	glomerular filtration rate
NP	natriuretic peptide
NPR	natriuretic peptide receptor
RAAS	renin-angiotensin-aldosterone system
RMIC	renal medullary interstitial cell
RPF	renal plasma flow
SNS	sympathetic nervous system
